# Risk factors for recurrence in pediatric urinary stone disease

**DOI:** 10.1007/s00467-024-06300-0

**Published:** 2024-01-25

**Authors:** Ferhan Demirtas, Nilgün Çakar, Zeynep Birsin Özçakar, Aykut Akıncı, Berk Burgu, Fatoş Yalçınkaya

**Affiliations:** 1 Department of Pediatrics, Ankara University School of Medicine, Ankara, Turkey; 2 Division of Pediatric Nephrology, Department of Pediatrics, Ankara University School of Medicine, Ankara, Turkey; 3 Department of Pediatric Urology, Ankara University School of Medicine, Ankara, Turkey

**Keywords:** Children, Urolithiasis, Risk factors, Metabolic disorder, Recurrence, Outcome

## Abstract

**Background:**

Children’s urinary system stones may develop from environmental, metabolic, anatomical, and other causes. Our objective is to determine the recurrence and prognosis, demographic, clinical, and etiological characteristics of children with urolithiasis.

**Methods:**

Medical records of patients were evaluated retrospectively. Patients’ demographic data and medical history, serum/urine biochemical and metabolic analysis, blood gas analysis, stone analysis, imaging findings, and medical/surgical treatments were recorded.

**Results:**

The study included 364 patients (male 187). Median age at diagnosis was 2.83 (IQR 0.83–8.08) years. The most common complaints were urinary tract infection (23%) and urine discoloration (12%). Sixty-two percent had a family history of stone disease. At least one metabolic disorder was found in 120 (88%) of 137 patients having all metabolic analyses: hypercalciuria was found in 45%, hypocitraturia in 39%, and hyperoxaluria in 37%. Anatomical abnormalities were detected in 18% of patients. Of 58 stones analyzed, 65.5% were calcium and 20.6% were cystine stones. Stone recurrence rate was 15% (55/364). Older age (> 5 years), family history of stone disease, stone size (≥ 5 mm), and urinary system anatomical abnormalities were significantly associated with stone recurrence (*p* = 0.027, *p* = 0.031, *p* < 0.001, and *p* < 0.001, respectively). In adjusted logistic regression analysis, stone size ≥ 5 mm (OR 4.85, 95% CI 2.53–9.3), presence of urinary system anatomical abnormalities (OR 2.89, 95% CI 1.44–5.78), and family history of stone disease (OR 2.41, 95% CI 1.19–4.86) had increased recurrence rate.

**Conclusions:**

All children with urolithiasis should be evaluated for factors affecting stone recurrence. Children at higher risk of recurrence need to be followed carefully.

**Graphical abstract:**

**Figure:**
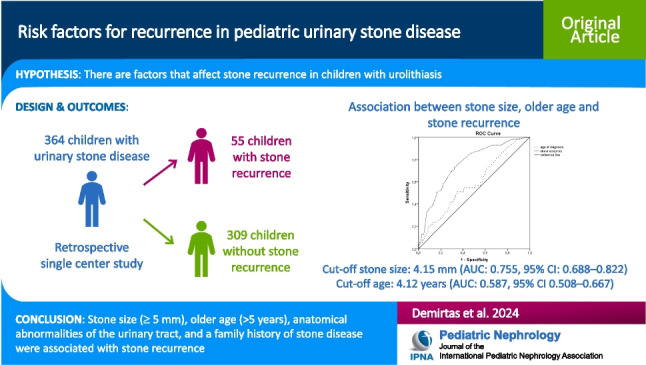
A higher resolution version of the Graphical abstract is available as Supplementary information

**Supplementary Information:**

The online version contains supplementary material available at 10.1007/s00467-024-06300-0.

## Introduction

Urinary stone disease is characterized by the presence of stones in the kidney, ureter, bladder, urethra, and/or calcification in the kidney. The frequency of the disease in children has increased from 4–6% to 10% in recent years [1–4]. In addition to lifestyle and dietary changes, more frequent use of imaging modalities is effective in this increase [2, 5]. Environmental, metabolic, anatomical, infectious, nutritional, and genetic causes play a role in the etiology of the disease [6–9]. Anatomical and metabolic causes are more common in children, resulting in higher stone recurrence rates and complications such as loss of kidney function [10, 11]. The European Association of Urology advises high-risk individuals to have 24-h urine samples examined to determine their metabolic risk and receive targeted medication to reduce their risk of recurrence [12]. Nevertheless, the likelihood of recurrence in children is not well understood.

In this study, we aimed to determine the demographic, clinical, and etiological characteristics, stone recurrence rate, factors affecting stone recurrence, and prognosis of children with urolithiasis.

## Patients and methods

We retrospectively reviewed the medical records of patients under 18 years of age who were admitted to the Department of Pediatric Nephrology in our hospital with urolithiasis between 2013 and 2018 and followed for at least 6 months. The study was approved by the Ankara University Faculty of Medicine Clinical Research Ethics Committee (18–1200-18. 11.12.2018).

Demographic data of the patients (gender, age at diagnosis), family history of stone disease, history of urinary tract infection, presenting symptoms, urinalysis, urine culture, spot urine and/or 24-h urine calcium, oxalate, citrate, uric acid, cystine, amino acids, urine stone analysis, imaging findings (localization, size, number of stones, urinary tract abnormalities), and medical and surgical treatments were recorded.

Serum biochemical analyses were performed in all patients. Serum parathormone (PTH) and vitamin D levels were measured in patients with hypercalcemia.

Spot urinalysis and biochemical tests were studied with the ion selective electrodes (IES) indirect method, blood gas analysis with the ABL800 Flex device, calcium in spot and 24-h urine with arsenazo, uric acid with uricase, creatinine with Jaffe, and urine amino acids with chromatographic methods. The analysis of the stones that could be obtained by spontaneous passage, extracorporeal shock wave lithotripsy (ESWL), percutaneous nephrolithotomy (PCNL), and/or open surgery was made by X-ray diffraction at the Institute of Mineral Inspection and Research Laboratory, Ankara, Turkey. In spot urinalysis, the number of erythrocytes and leukocytes less than five in the microscopic examination of urine was defined as microscopic hematuria and pyuria, respectively. Normal urinary calcium, citrate, oxalate, uric acid, cystine, and citrate values by age are evaluated according to Harvey et al. [1]. Urine metabolic tests were performed at the earliest period when there was no macroscopic hematuria and/or urinary tract infection, and the patient was not receiving intravenous fluids or drug therapy.

Urine metabolic analyses were requested for almost all patients. However, not all examinations could be completed in patients who refused to urinate, gave insufficient amounts of urine, or did not want to collect 24-h urine. Patients who had an analysis of urinary calcium, oxalate, uric acid, cystine, citrate, and urine amino acids defined the group with a complete metabolic analysis. Due to the possibility of more than one metabolic disorder, patients who did not complete all their examinations were not included in the patient group with complete urinary metabolic analysis performed.

For all patients, the stones were documented by renal ultrasonography (USG) and/or spontaneous stone passage. A sonographic examination was performed to determine stone growth or stone formation during follow-up.

Patients with stone recurrence were examined every 6 months, or more frequently if there were complaints. Patients with stones below 5 mm, without an increase in the number or size of stones, have wider examination intervals during follow-up.

### Statistical analyses

Statistical analyses were performed with IBM SPSS 22. Continuous variables are shown as mean and standard deviation or median with quartiles (25th and 75th percentiles); categorical variables are shown as numbers of cases and percentages. A chi-square and Fisher’s exact tests were used to compare proportions. In the comparison of the two groups, the variables were analyzed by the ındependent sample *T* test or the Mann–Whitney *U* test according to their distribution. The receiver operating characteristic (ROC) curve was used to calculate the specificity and sensitivity of the diagnostic tests and to calculate the cut-off values to be used for the tests. Logistic regression analyses were used to evaluate odds ratios (ORs) and adjusted ORs. *p* < 0.05 was accepted as the statistical significance limit.

## Results

The study included 364 patients (187 males). The median age at diagnosis was 2.83 (IQR 0.83–8.08) years. The most common complaints at admission were urinary tract infection (UTI) (23%), urine discoloration (12%), and abdominal pain (11%). Some patients had multiple complaints. Fifty-five percent of the patients had a history of UTI, and 62% had a family history of stone disease. Of our patients, 5 were born prematurely, 7 were immobile, and 6 received topiramate. Sixty-seven percent of the patients had microscopic hematuria, and 69% had pyuria at admission. The demographic and clinical features of patients are presented in Table 1.

**Table 1:** Demographic and clinical features of the patients

Demographic features	*n* = 364, *n* (%)	Median (IQR 25–75)
Age at diagnosis (year)		2.83 (0.83–8.08)
Female Male	177 (49) 187 (51)	3.16 (0.91–8.70) 2.50 (0.66–7.25)
Group—all metabolic analyses performed	*n* = 137, *n* (%)	
Age at diagnosis (year)		3.83 (1.08–7.83)
Female Male	68 (49.6) 69 (50.4)	2.79 (0.77–8.35) 3.91 (1.66–7.12)
Signs and symptoms		
UTI Discoloration of urine Abdominal pain Vomiting Restlessness Flank pain Dysuria/crying while urinating Seeing particles in urine Urinary system anomaly^†^ Pass urinary stone None (incidental)^‡^	95 (23) 51 (12) 46 (11) 32 (8) 27 (6) 22 (5) 21 (5) 14 (3) 11 (3) 11 (3) 91 (22)	
History		
History of UTI Family history of urinary stone disease Consanguineous marriage^§^ Antenatal hydronephrosis History of immobilization Antiepileptic (topiramate) use Premature birth	202 (55) 224 (62) 60 (24) 9 (2) 7 (2) 6 (2) 5 (1.4)	
Physical examination		
Body weight		
< 3 percentile > 97 percentile Phimosis/labial fusion Costovertebral angle tenderness Hypertension Cryptorchidism Hypospadias	22 (6) 9 (2.5) 19 (5) 18 (5) 3 (1) 3 (1) 1 (0.3)	
Urinalysis		
Pyuria Proteinuria (prot/cre) (*n* = 258) Microscopic hematuria	91 (25) 58 (22) 70 (19)	
Stone diagnosis		
USG Spontaneous stone passage	353 (97) 11 (3)	

*UTI* urinary tract infection, *USG* ultrasonography

^†^Hypospadias, cryptorchidism, antenatal hydronephrosis ^‡^It includes constipation, prolonged jaundice, single umbilical artery, increased liver enzymes, hemangioma, syndromic appearance, cystic fibrosis, hereditary spherocytosis, gastritis, hypercalcemia, hepatomegaly, pheochromocytoma, antiepileptic use, rickets, ventriculoperitoneal shunt, hypoparathyroidism, hyperparathyroidism, diabetes insipidus, calciuria or proteinuria in spot urine analysis during healthy child follow-up, positive family history of urinary stone disease ^§^Consanguinity was questioned in 249 patients

Biochemical analysis of serum was abnormal in 17 patients. Of these patients, 2 had high creatinine and 10 (2.7%) had hypercalcemia. Seven patients with hypercalcemia were diagnosed with idiopathic hypercalcemia, one each with Di-George syndrome, hyperparathyroidism, and vitamin D toxicity. The final diagnosis of patients with abnormal serum biochemical analysis is shown in Table 2.

**Table 2:** Abnormal serum biochemical analyses and associated conditions

Analysis of serum (n)	*n* (%)	Disease
Elevated creatinine (364) Hypercalcemia (364)	2 (0.5) 10 (2.7)	Primary hyperoxaluria DiGeorge syndrome in 1 patient Hyperparathyroidism in 1 patient Vitamin D toxicity in 1 patient (> 600 IU) Idiopathic hypercalcemia in 7 patients
Blood gas analysis (241)		
Metabolic acidosis Metabolic alkalosis Hyperuricemia (364) Elevated vitamin D (101)	1 (0.4) 1 (0.4) 1 (0.2) 2 (2)	Distal RTA Bartter syndrome Lesch–Nyhan syndrome 25 (OH) D level (> 50 IU)

*RTA* renal tubular acidosis

Full metabolic analysis of urine was performed in 137 (37%) of the patients. The gender and ages of the patients in this group were similar to the whole cohort. At least one metabolic disorder was detected in 120 of them (88%). Some patients had more than one metabolic disorder. Hypercalciuria, the most common metabolic abnormality, was detected in 61 (45%) of the patients. Fifty-seven (93%) of them were normocalcemic, and 4 were hypercalcemic. Forty-five (79%) of 57 normocalcemic patients had idiopathic hypercalciuria. Hypocitraturia was found in 54 (39%), hyperoxaluria in 51 (37%), hyperuricosuria in 30 (22%), and cystinuria in 12 (8%) of the patients. Two of the patients with hyperoxaluria were diagnosed as having primary hyperoxaluria type 1 (PH1) (Table 3).

**Table 3:** Biochemical analysis of urine in patients who had all metabolic analyses performed

Analysis of urine (*n* = 137)	*n* (%)
Hypercalciuria Hypocitraturia Hyperoxaluria Hyperuricosuria Cystinuria Normal	61 (45) 54 (39) 51 (37) 30 (22) 12 (8) 17 (12)

In some patients, more than one metabolic disorder has been found

Urinary system USG was performed on all patients. Forty-two percent of the patients had a single stone on their USG. Most of the stones (96%) were located in the kidney (Table 4). The mean stone size was 4.67 ± 3.62 mm. Nephrocalcinosis was detected in 24 (7%) patients. Eleven of these patients also had stones in the urinary system. The median age at diagnosis of patients with nephrocalcinosis was 2.41 years (IQR 0.91–8.64), and complete urinary metabolic analysis was performed to all patients. Hypercalciuria was detected in 16 (67%) of the patients, hyperoxaluria in 7 (29%), hypocitraturia in 6 (25%), uricosuria in 5 (21%), and cystinuria in 1 (4%). Nephrocalcinosis of 8 patients resolved spontaneously. Metabolic disorder was detected in 3 of these 8 patients. Hypercalciuria was detected in 3 patients, uricosuria in 2, hyperoxaluria in 1, and hypocitraturia in 1.

**Table 4:** Imaging findings of patients

	Finding	*n* (%)
Ultrasonography(*n* = 364)	Stone	364 (100)
	Kidney	348 (95.6)
	Both kidneys One kidney	220 (60.4) 128 (35.1)
	Ureter	14 (3.8)
	Bladder	1 (0.3)
	Urethra	1 (0.3)
	Nephrocalcinosis Hydronephrosis Atrophic kidney Cyst	24 (7) 39 (11) 10 (3) 2 (1)
Voiding cystourethrography (*n* = 64)	VUR	9 (14)
Computed tomography (*n* = 33)	Stone Atrophic kidney Mass	24 (73) 2 (6) 1 (3)
DMSA scintigraphy (*n* = 82)	Decrease in kidney function / renal scarring	26 (32)
MAG3 scintigraphy (*n* = 11)	UPJ obstruction UVJ obstruction	10 (91) 1 (9)
Renal vein Doppler sonography^¶^ (*n* = 6)	Nutcracker syndrome	2 (33)
Intravenous pyelography (*n* = 1)	Normal	1 (100)

*DMSA* dimercaptosuccinic acid, *MAG3* mercaptoacetyltriglycine, *VUR* vesicoureteral reflux, *UPJ* ureteropelvic junction, *UVJ* ureterovesical junction

^¶^Performed due to proteinuria

Anatomical abnormalities (hydronephrosis, atrophic kidney, ureteropelvic junction obstruction, vesicoureteral reflux, and ureterovesical junction obstruction) were detected in 65 (18%) patients. Details of the image findings are shown in Table 4.

Of the 58 stones analyzed, 38 (65.5%) were calcium stones and 12 (20.6%) were cystine stones. The results of the stone analysis are shown in Table 5.

**Table 5:** Stone analysis

Stone analysis	*n* = 58, *n* (%)
Calcium stone	38 (65.5)
Calcium oxalate Calcium phosphate Mix calcium	30 (79) 1 (2.6) 7 (18.4)
Cystine stone	12 (20.6)
Struvite stone	3 (5)
Uric acid stone	2 (3)
Xanthine stone	2 (3)
Quartz stone	1 (2)

The median follow-up period of the patients was 17 months (IQR 7.00–31.75). Seventy-one percent of 226 patients with a stone size < 5 mm and 54% of 125 patients with ≥ 5 mm were stone-free at the last visit. A statistically significant inverse correlation was found between stone size and stone-free rates (*p* = 0.002).

Of the 195 patients followed for more than 24 months, 135 (69%) were stone-free at the last visit. The stones disappeared spontaneously in 86 (64%) of these patients, dropped out in 12 (9%), and 37 (27%) became stone-free with surgical treatment. The stone sizes of these patients were 3.14 ± 1.83 mm, 3.92 ± 1.23 mm, and 7.92 ± 4.52 mm, respectively. It was found that if the stone size was < 3.45 mm, spontaneous disappearance was significantly higher, with a 72.4% sensitivity and 52.4% specificity (*p* < 0.05). The stone-free rate was 69% for those with a single stone and 62% for those with multiple stones. However, the number of stones was not associated with being stone-free at the last visit.

Overall, the stone recurrence rate was 15% (55/364). The first stone recurrence was observed median 30 months (IQR 22–48) and occurred in 50.9% of the patients within 30 months after the initial stone diagnosis. The highest stone recurrence rate (75%) was found in patients with cystine stones. However, stone recurrence in patients with complete urinary metabolic analysis was not found to be higher in patients with metabolic disorders than those without metabolic disorders (*p* > 0.05) (Table 6).

**Table 6:** Risk factors for stone recurrence

Characteristics (*n* = 364)	Recurrence, *n* (%)	Non-recurrence, *n* (%)	*p*
Age of diagnosis (year)			**0.027**
≤ 5 years (*n* = 227) > 5 years (*n* = 137)	27 (12) 28 (20)	200 (88) 109 (80)	
History of UTI			0.056
Absent (*n* = 162) Present (*n* = 202)	18 (11) 37 (18)	144 (89) 165 (82)	
Family history of urinary stone disease			**0.031**
Absent (*n* = 140) Present (*n* = 224)	14 (10) 41 (18)	126 (90) 183 (82)	
Stone size			** < 0.001**
< 5 mm (*n* = 239) ≥ 5 mm (*n* = 125)	17 (7) 38 (30)	222 (93) 87 (70)	
Stone number			0.119
Single stone (*n* = 154) Multiple stones (*n* = 210)	18 (12) 37 (18)	136 (88) 173 (82)	
Anatomical abnormalities			** < 0.001**
Absent (*n* = 299) Present (*n* = 65)	34 (11) 21 (32)	265 (89) 44 (68)	
Metabolic risk factors (*n* = 137)			1.000
Absent (*n* = 17) Present (*n* = 120)	5 (29) 34 (28)	12 (71) 86 (72)	

**Fig. 1:**
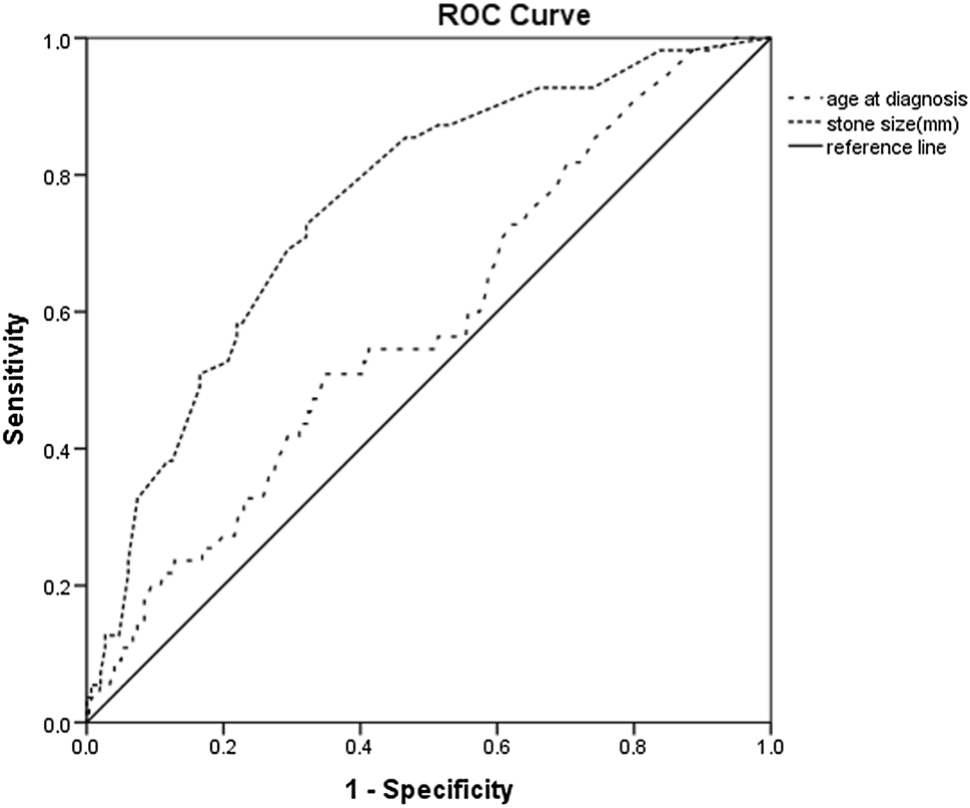
ROC curve for age at diagnosis and stone size with stone recurrence

It was observed that stone recurrence increased with age (*p* = 0.039), and the cut-off value for age was 4.12 years (AUC 0.587, 95% CI 0.508–0.667) (55% sensitivity and 59% specificity) (Fig. 1). Family history of stone disease, microscopic hematuria, and pyuria at presentation were risk factors for stone recurrence (*p* = 0.031, *p* = 0.017, and *p* = 0.001, respectively) (Table 6). Stone size was strongly associated with stone recurrence (*p* < 0.001) (Table 6), and the cut-off stone size was found to be 4.15 mm (AUC 0.755, 95% CI 0.688–0.822) (73% sensitivity and 68% specificity) (Fig. 1). Stone recurrence was also high in patients with urinary system anatomical abnormalities (*p* < 0.001). Adjusted logistic regression analysis also showed that children with a stone size ≥ 5 mm had a higher risk of recurrence (OR 4.85, 95% CI 2.53–9.3). Urinary system anatomical abnormalities (OR 2.89, 95% CI 1.44–5.78) and a family history of stone disease (OR 2.41, 95% CI 1.19–4.86) were associated with increased stone recurrence as well.

One hundred twenty patients (33.1%) had antibiotic prophylaxis for recurrent urinary tract infections, while 130 patients (36%) received one or more medications for urinary stone disease (Table 7). All 12 patients with cystinuria were using cystine-binding thiols (alpha-mercaptopropionyl-glycine in 11 patients, d-penicillamine in 1 patient), and urine alkalizing medication (potassium citrate). Patients with PH1 were receiving pyridoxine and potassium citrate treatments. Surgical intervention was performed on 100 individuals (27%) of the total. All 12 patients with cystine stones underwent surgical intervention (ESWL, ureteroscopy (URS), PCNL, or open surgery). Kidney function regressed in the unilateral kidneys of four patients. Unilateral nephrectomy was performed in 3 patients due to non-functioning kidneys (1 cystinuria, 1 xanthinuria, and 1 hypercalciuria). Two patients with PH1 developed chronic kidney disease (CKD) during the follow-up.

**Table 7:** Medical and surgical treatments of patients

Medical treatments	*n* = 130, *n* (%)
Potassium citrate Hydrochlorothiazide Pyridoxine Allopurinol Alpha-mercaptopropionyl glycine d-Penicillamine	91 (70) 41 (31.5) 14 (10.8) 2 (1.5) 11 (8) 1 (0.8)
Surgical interventions	*n* = 100, *n* (%)
ESWL	70 (70)
1 time More than 1 time	42 (42) 28 (28)
URS PCNL Open surgery	46 (46) 16 (16) 6 (6)

Some patients had more than one medical and surgical treatment

*ESWL* extracorporeal shock wave lithotripsy, *PCNL* percutaneous nephrolithotomy, *URS* ureteroscopy

## Discussion

Urinary stone disease is rarely seen in children compared to adults, but its frequency has increased in recent years due to lifestyle and dietary changes and more frequent use of imaging modalities [2–5]. The mechanism of stone formation in the urinary system is not fully known. It was reported that approximately 50–75% of urinary stones occur as a result of metabolic abnormalities in children, so the rate of stone recurrence and kidney damage is high [6–10]. Therefore, detailed research on the primary cause and effective treatment is recommended in children with urolithiasis.

Urinary stone disease occurs at any age and in approximately equal proportions in both sexes in children, as in our study [5, 7, 11, 13–15]. Renal colic and macroscopic hematuria, which are the most common presenting signs and symptoms of urinary stone disease in adults, are rare in children. Children generally do not have any complaints about stones [9, 16, 17]. The most common reasons for referral in children are urinary tract infections (36–37%) and abdominal pain (32–58%) [14, 16, 18]. Besides, stone disease is detected incidentally in 11–24% of the patients in childhood [14, 16, 18]. The most common complaint of our patients was UTI, seen in more than one-fifth of the patients, followed by discoloration of the urine and abdominal pain whereas 22% of the patients were referred due to the incidental detection of stones on USG. The family history of urinary stone disease in children with urolithiasis has been reported to be 40–85% [11, 14, 18]. Consistent with this range, 62% of our patients had a family history of stone disease. Inflammatory bowel disease, cystic fibrosis, prematurity, long-term immobilization, and antiepileptic (topiramate) use are among the risk factors for urinary stone disease [1, 14]. Five of our patients were born prematurely, 7 were immobile, and 6 were taking topiramate. Obesity, which is reported to be prone to stone formation in adults, has not been reported to increase stone risk in children, as in our patient group [11, 14, 16].

The rate of metabolic disorders of stone etiology in children varies between 30 and 93%, and these are generally mixed disorders [7, 11, 13, 14, 16]. Metabolic disorders were found in 88% of our 137 patients who underwent complete metabolic analysis, and more than one metabolic disorder was detected in 55% of them. As in our patient group, hypercalciuria was the most common metabolic disorder previously reported (34–43%) [7, 14, 16]. In the present study, 93% of patients with hypercalciuria were normocalcemic, and the majority (79%) of them were idiopathic, similar to those in the literature [19]. Hypercalcemic hypercalciuria was detected only in 4 patients. Of 51 (37%) patients with hyperoxaluria, 2 were diagnosed with PH1, and these patients developed CKD at follow-up. It has been reported that hypocitraturia is detected in 15–70% of patients, and most cases are accompanied by other metabolic disorders [11, 13, 14]. Some studies have also reported that hypocitraturia is the most common metabolic disorder [20, 21]. In our patients, the second most common metabolic disorder was hypocitraturia, and 2/3 of the patients with hypocitraturia were accompanied by other metabolic disorders. Cystine stones, which account for 2–8% of kidney stones in children [22], constituted 8% of our patients who underwent complete metabolic analysis. The prevalence may vary according to different populations; the high percentage of cystine stones in our research may result from the high frequency of consanguineous marriage. In studies conducted in our country, the frequency of cystine stones in children under 1 year of age has been reported as 6–17% of all metabolic stone diseases [7, 23].

Ultrasonography, which has no radiation effect and shows the anatomical abnormality, location, number, and size of the stones, is widely used in the diagnosis of urinary stone disease [1, 14, 18]. Ninety-seven percent of our patients were diagnosed with urinary stones by USG, and it was used in the follow-up of all patients. Although computed tomography (CT) is a very sensitive procedure in the diagnosis of stones, it is not recommended for every patient due to radiation exposure, except in suspicious cases. It was used on only 9% of our patients for advanced examination and treatment process.

About three-quarters of urinary stones form in the kidneys [7, 14, 24]. Bladder stones that occur less frequently can form in the bladder or fall into the bladder from the upper urinary tract. Stones formed in the bladder can occur after malnutrition, especially protein-poor nutrition, early start of carbohydrate feeding, low milk supply in newborns, the presence of a foreign body in the bladder, or bladder surgery [1, 25]. Only 0.3% of our patients had bladder stones.

The number of stones analyzed in children is low; this is due to the fact that the stones are passed at home, and families do not know the importance of stone analysis [14, 18]. The most common mineral in stone analysis is calcium, and most of these are calcium oxalate stones [11, 14, 16, 18, 24, 26, 27]. Of the 58 stones analyzed in this study, 65.5% contained calcium, and more than three-quarters of these stones were calcium oxalate stones.

The priority in the treatment of urinary stone disease in children is to increase fluid intake and avoid excessive salt consumption. Drug therapy is directed at the primary metabolic disorder. Due to metabolic disorders, the most commonly used drugs are reported to be potassium citrate and hydrochlorothiazide [18, 28], as in our study. It was reported that surgery was performed in the treatment of urinary stones in 24–38% of children, and 12–15% of patients underwent ESWL [14, 18, 29]. In the present study, 27% of patients underwent surgical intervention, of which 70% underwent ESWL, 46% underwent URS, 16% underwent PCNL, and 6% underwent open surgery. Among patients with metabolic disorders, patients with cystinuria constituted the group in which surgical intervention was most frequently applied. As expected, stone recurrence in our patients increased the need for medical and/or surgical interventions (*p* < 0.001).

It has been reported that urinary tract stones < 5 mm disappear spontaneously, 50% of the stones between 5 and 7 mm fall spontaneously, and stones larger than 7 mm generally require surgical intervention [30]. When we evaluated the stone-free status at the last visit according to the stone size of patients, it was found that 71% of those with stones < 5 mm and 54% of those with stones ≥ 5 mm were stone-free. A statistically significant inverse relationship was found between stone size and stone-free status at the last visit (*p* = 0.002). A stone size of 3.45 mm was found to have high sensitivity (72.4%) and specificity (52.4%) in the disappearance of stones (*p* < 0.05).

The rate of stone recurrence, which was 15% in our patient group, has been reported to be 16–29% in the literature [13, 19, 30, 31]. The first recurrence occurred in 50.9% of our patients within 30 months after the initial stone diagnosis. Tasian et al. reported that symptomatic recurrence was observed in almost 50% of children within 3 years after the first stone [32]. The most frequently recurrent stones are cystine stones, with a rate of 60–70% [19, 33]. The stone recurrence rate in our patients with cystinuria was 75%. In the patient group in which all metabolic analyses were performed, recurrence rates were not different between those with and without metabolic disorders (*p* > 0.05). This result may be due to the fact that 88% of the patients in this group had metabolic abnormalities and the number of patients without metabolic abnormalities was small.

Anatomical abnormalities of the urinary system have been reported as another risk factor for the formation and recurrence of urinary stones [14, 29]. Vaughan et al. reported that the risk of symptomatic recurrence increased with age, male gender, body mass index, family history of stones, incidental stones on imaging before the first confirmed stone attack, the number of kidney stones, and stones with a diameter of 3–6 mm [34]. Another article showed that older children, higher BMI, and higher stone burden were associated with stone recurrence in univariate analysis. Multivariate Cox regression analysis also showed that the risk of recurrence is higher in children with a high body mass index and a stone burden > 2 cm^3^ [35]. In our study, being older than 5 years, having a family history of stone disease, having microscopic hematuria and pyuria at presentation, and having a stone size greater than 5 mm were also associated with stone recurrence. Adjusted logistic regression analysis also showed that children with a stone size ≥ 5 mm (OR 4.85, 95% CI 2.53–9.3), urinary tract anatomical abnormalities, and a family history of stone disease were associated with the risk of stone recurrence (OR 2.89, 95% CI 1.44–5.78, and OR 2.41, 95% CI 1.19–4.86, respectively).

Despite medical treatment, high recurrence rates require frequent surgical intervention, and patients are at high risk for kidney damage. The risk of developing CKD is especially high in primary hyperoxaluria, cystinuria, and xanthinuria, where the risk of stone recurrence is high.

This study had some limitations. Firstly, the study was conducted retrospectively in a single center, and secondly, complete metabolic analyses were not performed in all patients.

In summary, a high percentage of urinary stone disease in children is caused by metabolic disorders such as hypercalciuria and hypocitraturia. Stone size was important in the disappearance and recurrence of stones in the absence of metabolic disorders, such as cystinuria, xanthinuria, and primary hyperoxaluria, and urinary system anatomical anomalies. Our study also showed that older age (> 5 years), anatomical abnormalities of the urinary tract, and a family history of stone disease were associated with stone recurrence.

### Supplementary Information

Below is the link to the electronic supplementary material.

Graphical abstract (PPTX 90 KB)

## Data Availability

The dataset generated during and/or analyzed during the current study is available from the corresponding author on reasonable request.
