# Supplementary material for: Risk factors for recurrence in pediatric urinary stone disease
Source: Pediatr Nephrol. 2024 Jan 25;39(7):2105–13. doi: 10.1007/s00467-024-06300-0 (PMC11147915; doi:10.1007/s00467-024-06300-0)
Supplement: Supplementary file 1 — Graphical abstract (PPTX 90 KB) [file 467_2024_6300_MOESM1_ESM.pptx]

## Slide 1
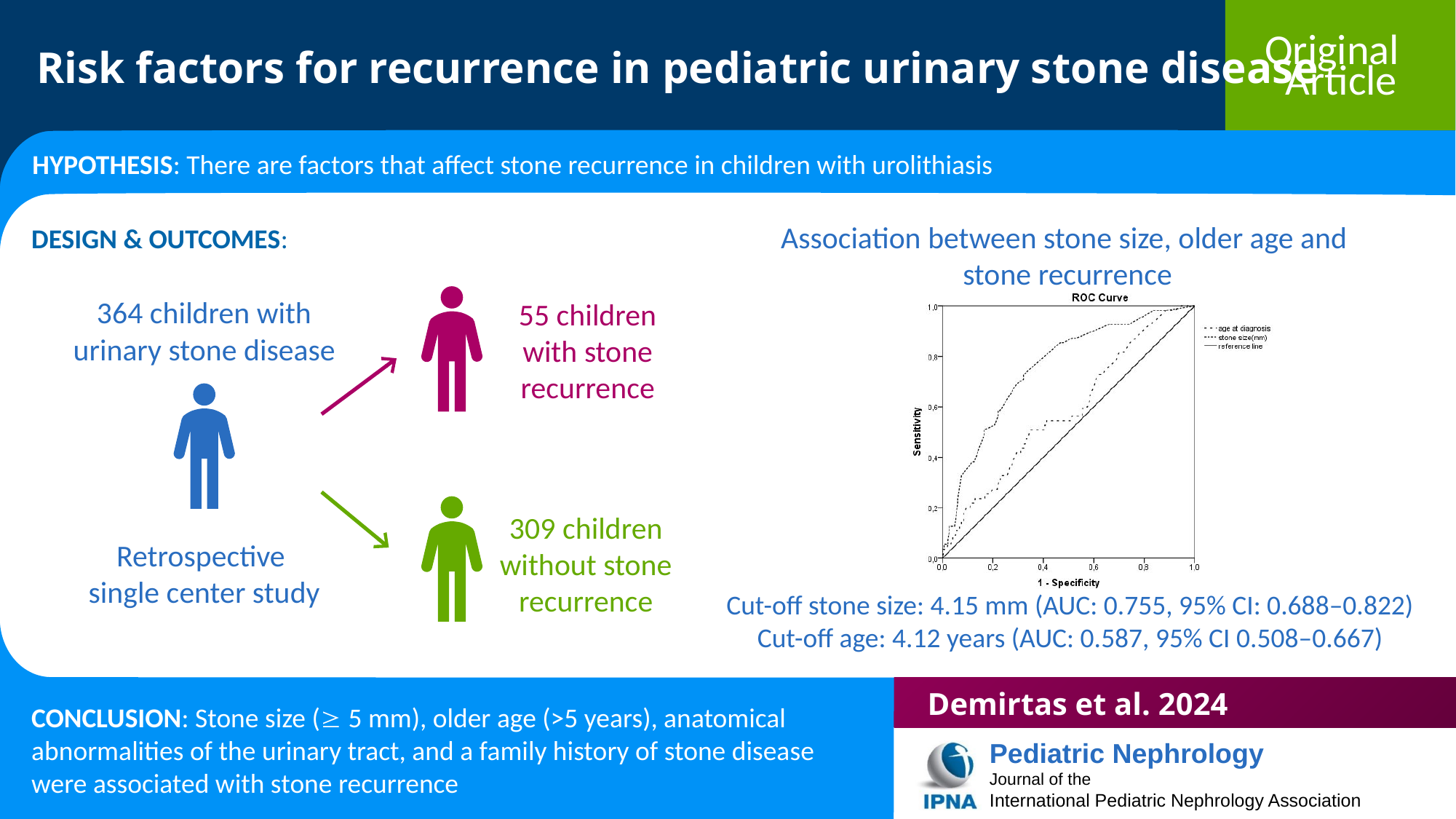

Risk factors for recurrence in pediatric urinary stone disease
HYPOTHESIS: There are factors that affect stone recurrence in children with urolithiasis
Association between stone size, older age and
stone recurrence
DESIGN & OUTCOMES:
364 children with urinary stone disease
55 children with stone recurrence
309 children without stone recurrence
Retrospective
single center study
Cut-off stone size: 4.15 mm (AUC: 0.755, 95% CI: 0.688–0.822)
Cut-off age: 4.12 years (AUC: 0.587, 95% CI 0.508–0.667)
Demirtas et al. 2024
CONCLUSION: Stone size (≥ 5 mm), older age (>5 years), anatomical abnormalities of the urinary tract, and a family history of stone disease were associated with stone recurrence
